# Lipid Classes and Fatty Acids in *Ophryotrocha cyclops*, a Dorvilleid from Newfoundland Aquaculture Sites

**DOI:** 10.1371/journal.pone.0136772

**Published:** 2015-08-26

**Authors:** Flora Salvo, Suzanne C. Dufour, Dounia Hamoutene, Christopher C. Parrish

**Affiliations:** 1 Department of Biology, Memorial University of Newfoundland, St. John's, NL, Canada; 2 Fisheries and Oceans Canada, Northwest Atlantic Fisheries Centre, St. John's, NL, Canada; 3 Department of Ocean Sciences, Memorial University of Newfoundland, St. John's, NL, Canada; Louisiana State University Health Sciences Center, UNITED STATES

## Abstract

A new opportunistic annelid (*Ophryotrocha cyclops)* discovered on benthic substrates underneath finfish aquaculture sites in Newfoundland (NL) may be involved in the remediation of organic wastes. At those aquaculture sites, bacterial mats and *O*. *cyclops* often coexist and are used as indicators of organic enrichment. Little is known on the trophic strategies used by these annelids, including whether they might consume bacteria or other aquaculture-derived wastes. We studied the lipid and fatty acid composition of the annelids and their potential food sources (degraded flocculent organic matter, fresh fish pellets and bacterial mats) to investigate feeding relationships in these habitats and compared the lipid and fatty acid composition of annelids before and after starvation. Fish pellets were rich in lipids, mainly terrestrially derived C_18_ fatty acids (18:1ω9, 18:2ω6, 18:3ω3), while bacterial samples were mainly composed of ω7 fatty acids, and flocculent matter appeared to be a mixture of fresh and degrading fish pellets, feces and bacteria. *Ophryotrocha cyclops* did not appear to store excessive amounts of lipids (13%) but showed a high concentration of ω3 and ω6 fatty acids, as well as a high proportion of the main fatty acids contained in fresh fish pellets and bacterial mats. The dorvilleids and all potential food sources differed significantly in their lipid and fatty acid composition. Interestingly, while all food sources contained low proportions of 20:5ω3 and 20:2ω6, the annelids showed high concentrations of these two fatty acids, along with 20:4ω6. A starvation period of 13 days did not result in a major decrease in total lipid content; however, microscopic observations revealed that very few visible lipid droplets remained in the gut epithelium after three months of starvation. *Ophryotrocha cyclops* appears well adapted to extreme environments and may rely on lipid-rich organic matter for survival and dispersal in cold environments.

## Introduction

Where finfish aquaculture is conducted, organic matter (e.g., uneaten feed and feces) is released in the environment and may accumulate on the seafloor, thereby influencing benthic communities and sediment biogeochemistry. Assessing the level of environmental perturbation from aquaculture activities is often based on the determination of sediment sulfide or redox levels [1–3], and/or on macrofaunal community composition or multivariate indices [4–8]. In some regions, visual monitoring of the seafloor has revealed that certain organisms are associated with organic enrichment at aquaculture sites [9–12]; monitoring these ‘indicator species’ is a desirable alternative to chemical or community-based surveys, particularly in deep waters and hard/heterogeneous substrates and where sediment sampling is difficult. In NL, off the east coast of Canada, drop camera surveys have identified two indicator taxa that are abundant beneath salmonid aquaculture cages: mat-forming filamentous bacteria (considered to belong to the genus *Beggiatoa*) and dorvilleid annelids forming Opportunistic Polychaete Complexes (OPC), which may consist of one or more species [13–15]. *Beggiatoa* are sulfur-oxidising bacteria that form conspicuous white mats at redox interfaces [16,17], while OPC (a term used in aquaculture regulation) are aggregates of opportunistic polychaetes that colonize organically enriched substrates. In NL, OPC were recently shown to consist of a single new species within the family Dorvilleidae, *Ophryotrocha cyclops* Salvo et al. 2014; this species was also found on whale bones in Greenland [18]. Dorvilleids often co-occur with *Beggiatoa*-like mats in environments with high levels of (often transient) organic matter such as hydrothermal vents [19–21], whale bones [22,23], or aquaculture sites [24–26].

Understanding the ecology and life traits of both OPC and associated mat-forming, *Beggiatoa*-like bacteria is essential in defining their potential role in organic matter remineralization under aquaculture sites [18,27] and for developing a coherent regulation policy. Some legislations use OPC or bacterial mat coverage thresholds as determining factors in allowing site restocking after fallowing [28–30]. However, we currently have a limited understanding of the interactions between OPC, *Beggiatoa-*like mats and organic matter at aquaculture sites. A trophic analysis of *Ophryotrocha cyclops* in NL aquaculture sites based on stable isotopes of nitrogen, carbon and sulfur suggested that these worms mostly consume flocculent matter (a mixture of fish pellets and feces at various stages of degradation, particulates from the water column, and microbes); in that study, the relative dietary importance of bacteria was uncertain, as bacteria were not isolated from flocculent matter [27]. A better discrimination of *O*. *cyclops* food sources and of the importance of bacteria in their diet can be achieved using lipid and fatty acid (FA) analysis, as some FA are specific biomarkers of particular organisms (e.g., some iso and anteiso saturated FA >C_15_ are bacterial indicators) [31–34]. Interestingly, a high concentration of lipid droplets was observed in the gut epithelial lining of *O*. *cyclops* [27]. These lipid droplets may result from the incorporation of lipid-enriched fish feed, and/or may constitute a natural mechanism for those organisms to survive in cold, deep environments when food becomes scarce.

Numerous studies focusing on aquaculture sites have used FA and lipid analyses to examine energy transfer between species, the spatial and temporal footprint of deposited organic matter, or the digestibility of fish feed [26, 34–39]. In these studies, lipid classes and FA were used as biomarkers of bacteria, copepods, zooplankton, diatoms, or terrestrial organic matter [33,34,40,41]. Given that fish pellets contain a mixture of terrestrial and marine oils [42], they are likely to have characteristic lipid and FA signatures, distinct from those of bacteria. Here, we test whether *Ophryotrocha cyclops* from a NL aquaculture site consumes bacteria and assimilates lipids derived from fish pellets or flocculent matter by comparing the lipid and FA profiles of the annelids and their potential food sources. A secondary objective was to follow changes in the lipid and FA content of specimens maintained in the laboratory without food to examine which FA are preferentially catabolized by these annelids.

## Materials and Methods

### Sampling sites

Permits to collect invertebrates for experimental purposes were obtained from Fisheries and Oceans Canada. High densities of OPC were found on the seafloor directly underneath aquaculture cages in Hermitage Bay, NL (47.65°, -56.24°) during a regulatory drop camera survey [43]. At this site, fish had been harvested one month before the survey (no fresh fish pellets were present in the environment) and sampling were performed. Dorvilleids were sampled on June 2^nd^, 2014 using a net (0.5 mm mesh) attached to the camera frame, which was dragged along the seafloor (60 m depth). Upon retrieval, the net was rinsed using seawater from the site and the worms were picked, transferred to glass vials containing cold seawater, and held on ice. Flocculent matter collected in the net was run through a sieve (0.5 mm mesh) and three replicates were collected in glass jars [all glass vials and jars used in this study were previously cleaned with 10% HCl and, along with GF/F filters (47 mm, 0.7 μm porosity), were burnt for 4 h at 450°C]. Sequencing of the 16S gene confirmed that annelids belonged to the species *Ophryotrocha cyclops* [18].

Upon returning to the laboratory, seawater from the site was filtered (0.7 μm GF/F) and placed into glass jars where *Ophryotrocha cyclops* were maintained, at 4°C, in the absence of food (to allow gut content evacuation and to follow eventual lipid degradation); filtered seawater was replaced every other day. Over the first two days, worms excreted a large quantity of mucus with suspended feces: this material was collected using a transfer pipette, placed into 2 vials and immediately frozen (-20°C); a subsample was filtered onto two GF/F filters prior to freezing. Dorvilleid samples were pooled because of their small size and the detection limits for lipid and FA analyses. A first set of dorvilleids (n = 95, consisting of 8 replicates) was processed for lipid and FA analysis after 2 days of gut clearance whereas another set (n = 27, consisting of 2 replicates) was kept in filtered sterile seawater for two additional weeks prior to processing. Although specimens were maintained for 3 months in the lab and used for microscopic observation, there were insufficient numbers of *O*. *cyclops* remaining after 13 days to allow for lipid and FA analysis.

As it was not possible to obtain a sample of bacterial mats upon field sampling, we attempted to culture bacteria from a sample of flocculent matter and seawater from the aquaculture site in an open jar maintained at 4°C. After 20 days, a filamentous white mat (corresponding to the visual description of *Beggiatoa*-like mats in the field) developed at the seawater surface, however no species determination was performed and several species may have been present in this mat. Two replicate samples of this mat were obtained using a sterile scalpel and immediately frozen.

Fish pellets were provided to us by the company operating the aquaculture site, and three replicates were analyzed to determine their composition.

### Lipid and fatty acid analyses

All samples were collected in 40 mL glass test tubes that had been rinsed three times with methanol followed by three rinses with chloroform. Samples were added to tubes, weighed, and covered with 8 mL of chloroform. The tube headspace was then filled with nitrogen and samples were capped, sealed and stored at -20°C.

Lipid extraction was performed as described previously [44]. Samples were homogenized in a 2:1 mixture of ice-cold chloroform: methanol and homogenized with a Polytron PCU-2-110 homogenizer (Brinkmann Instruments, Rexdale, Ontario, Canada). Chloroform extracted water was added to bring the ratio of chloroform:methanol:water to 8:4:3. Samples were sonicated for 4 to 10 min in an ice bath and centrifuged at 5000 rpm for 2 min. The bottom, organic layer was removed using a double pipetting technique, placing a long lipid cleaned Pasteur pipette inside a short one, to remove the organic layer without disturbing the top, aqueous layer. Chloroform was then added back to the extraction test tube and the entire procedure was repeated 3 times. All organic layers were pooled into lipid-cleaned vials and concentrated using a flash-evaporator (Buchler Instruments, Fort Lee, NJ).

Lipid class composition was determined using an Iatroscan Mark VI TLC-FID, silica coated Chromarods and a three-step development method [45]. The lipid extracts were applied to the Chromarods and focused to a narrow band using 100% acetone. The first development system was hexane:diethyl ether:formic acid (99.95:1:0.05). The rods were developed for 25 min, removed from the system for 5 min and replaced for 20 min. The second development was for 40 min in hexane:diethyl ether:formic acid (79:20:1). The final development system had two steps, the first was 100% acetone for two 15 min time periods, followed by two 10 min periods in chloroform:methanol:chloroform-extracted water (5:4:1). Before placing in each solvent system the rods were dried in a constant humidity chamber. After each development system the rods were scanned in the Iatroscan and the data collected using Peak Simple software (3.67, SRI Inc). The Chromarods were calibrated using standards from Sigma Chemicals (Sigma Chemicals, St. Louis, MO). Lipid classes identified were: hydrocarbons (HC), steryl esters/wax esters (SE/WE), ethyl esters (EE), methyl esters (ME), ethyl ketones (EK), methyl ketones (MK), glycerol ethers (GE), triacylglycerols (TAG), free fatty acids (FFA), alcohols (ALC), sterols (ST), acetone mobile polar lipids (AMPL) and phospholipids (PL).

For all samples, lipid extracts were transesterified using methylene chloride and Hilditch reagent for 1 h at 100°C. The fatty acid methyl esters (FAME) were analyzed on a HP 6890 GC FID equipped with an 7683 auto-sampler. The GC column was a ZB wax+ (Phenomenex, USA). The column length was 30 mm with an internal diameter of 0.32 mm. The column temperature began at 65°C and was held at this temperature for 0.5 min. Temperature then ramped to 195°C at a rate of 40°C.min^-1^, was held for 15 min and then ramped to a final temperature of 220°C at a rate of 2°C.min^-1^. This final temperature was held for 0.75 min. The carrier gas was hydrogen flowing at a rate of 2 mL.min^-1^. The injector temperature started at 150°C and ramped to a final temperature of 250°C at a rate of 120°C.min^-1^. The detector temperature stayed constant at 260°C. Peaks were identified using retention times from standards purchased from Supelco, 37 component FAME mix (Product number 47885-U), Bacterial acid methyl ester mix (product number 47080-U), PUFA 1 (product number 47033) and PUFA 3 (product number 47085-U). Chromatograms were integrated using the Varian Galaxie Chromatography Data System, version 1.9.3.2.y.

Some FA were combined into classes: "bacterial biomarker" consists of *i*15:0, *ai*15:0, 15:0, 15:1, *i*16:0, *ai*16:0, *i*17:0, *ai*17:0, 17:0, 17:1 and 16:1ω6 [31,32]; "bacterial including ω7 FA" includes the latter set ("bacterial biomarker") plus 18:1ω7 and 16:1ω7 [33,36]. “SAFA” is the sum of saturated FA; PUFA and MUFA are the sum of poly- and mono- (respectively) unsaturated fatty acids; while EPA (20:5ω3) and DHA (22:6ω3) are the FA biomarkers of diatoms and dinoflagellates, respectively [33,40]. “AA” (20:4ω6) corresponds to arachidonic acid, while the "ω3" and "ω6" classes sum the omega-3 FA and omega-6 FA from a sample.

### Statistical analyses

Differences in FA and lipid composition between groups of worms experiencing different periods of starvation were not evaluated statistically because of the low level of replication in the starved samples (n = 2).

We used multivariate analysis to compare the FA composition of potential food sources and worms experiencing different periods of starvation, using PRIMER 6.0 [46,47] and PERMANOVA+ for PRIMER [48]. All available data were considered, and not transformed. First, a principal components analysis (PCA) was run. Then, clustering was performed. Similarity and dissimilarity within categories (i.e., food sources or worms) were explored on the untransformed matrix using Bray-Curtis distances with SIMPER and PERMANOVA procedures [47,48]. The PERMANOVA pairwise comparison was performed using the Monte Carlo correction, given the low number of sample replicates and the limited number of possible permutations [48].

### Thin sectioning and microscopy

Specimens of *Ophryotrocha cyclops* were fixed in 2.5% glutaraldehyde at different times: upon collection in the field, and after 13 days, five weeks and three months of laboratory maintenance in filtered seawater, with three individuals fixed at each time.

Dorvilleids were sectioned into anterior, median and posterior fragments, dehydrated in an ascending ethanol series and separately embedded in Epon resin. Thin (1 μm) sections from the median portion of one individual from each sampling time were stained with 1% toluidine blue and imaged with a Zeiss Axioscope.

## Results

Abbreviations used for lipid and FA classes are listed in Table 1. Unless indicated otherwise, results are presented as average proportions of total lipids or FA within samples ± standard errors.

**Table 1:** Lipid and Fatty Acid Classes. Abbreviations used, with some of the biological processes they indicate.

	Abbreviation	Indicator	Reference
**Lipid Classes**			
Triacylglycerols	TAG	Storage	65
Free Fatty Acids	FFA	Breakdown of TAG & PL, degraded products	38
Alcohols	ALC	Degradation of WE	
Sterols	ST	Membrane constituents	
Acetone Mobile Polar Lipids	AMPL	Breakdown of TAG	
Phospholipids	PL	Membrane constituent	
**Fatty Acid Classes**			
*i*15:0, *ai*15:0, 15:0, 15:1, *i*16:0, *ai*16:0, *i*17:0, *ai*17:0, 17:0, 17:1 and 16:1ω6	Bacterial markers	Sum of bacterial markers	31,32
18:1ω7		Bacterial marker	33
16:1ω7 and 18:1ω7		Sulfur oxidizing bacterial markers	54
Bacterial fatty acids +16:1ω7 and 18:1ω7	Bacterial marker including ω7	Bacterial markers	33,36,56
Sum of Omega 3 fatty acids	ω3		
Sum of Omega 6 fatty acids	ω6		
Saturated fatty acids	SAFA		
Monounsaturated fatty acids	MUFA		
Polyunsaturated fatty acids	PUFA		
PUFA/SAFA	PUFA/SAFA	Carnivory	67
22:6ω3 (Docosahexaenoic acid)	DHA	Dinoflagellate marker	40
20:5ω3 (Eicosapentaenoic acid)	EPA	Diatom marker	33
DHA/EPA or high 18:1ω9/18:1ω7 ratio		Carnivory	33,41
20:1ω9, 22:1ω9, 22:1ω11		Zooplankton markers	33,34

### Lipid and fatty acid composition of the potential food sources

**Fig 1:**
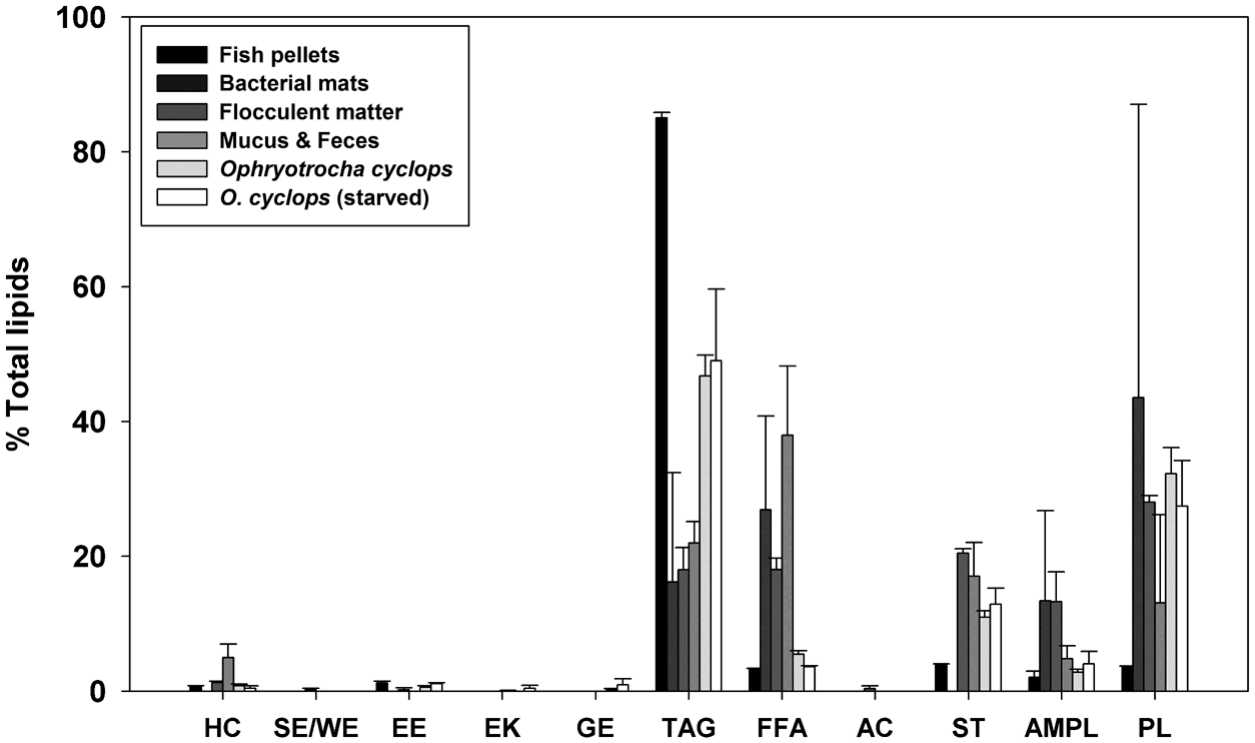
Lipid Class Composition (% of total lipid) of Samples from the Aquaculture Site. Error bars indicate SE. (HC: hydrocarbons, SE/WE: steryl esters/wax esters, EE: ethyl esters, ME: methyl esters, EK: ethyl ketones, MK: methyl ketones, GE: glycerol ethers, TAG: triacylglycerols, FFA: free fatty acids, ALC: alcohols, ST: sterols, AMPL: acetone mobile polar lipids and PL: phospholipids).

**Fig 2:**
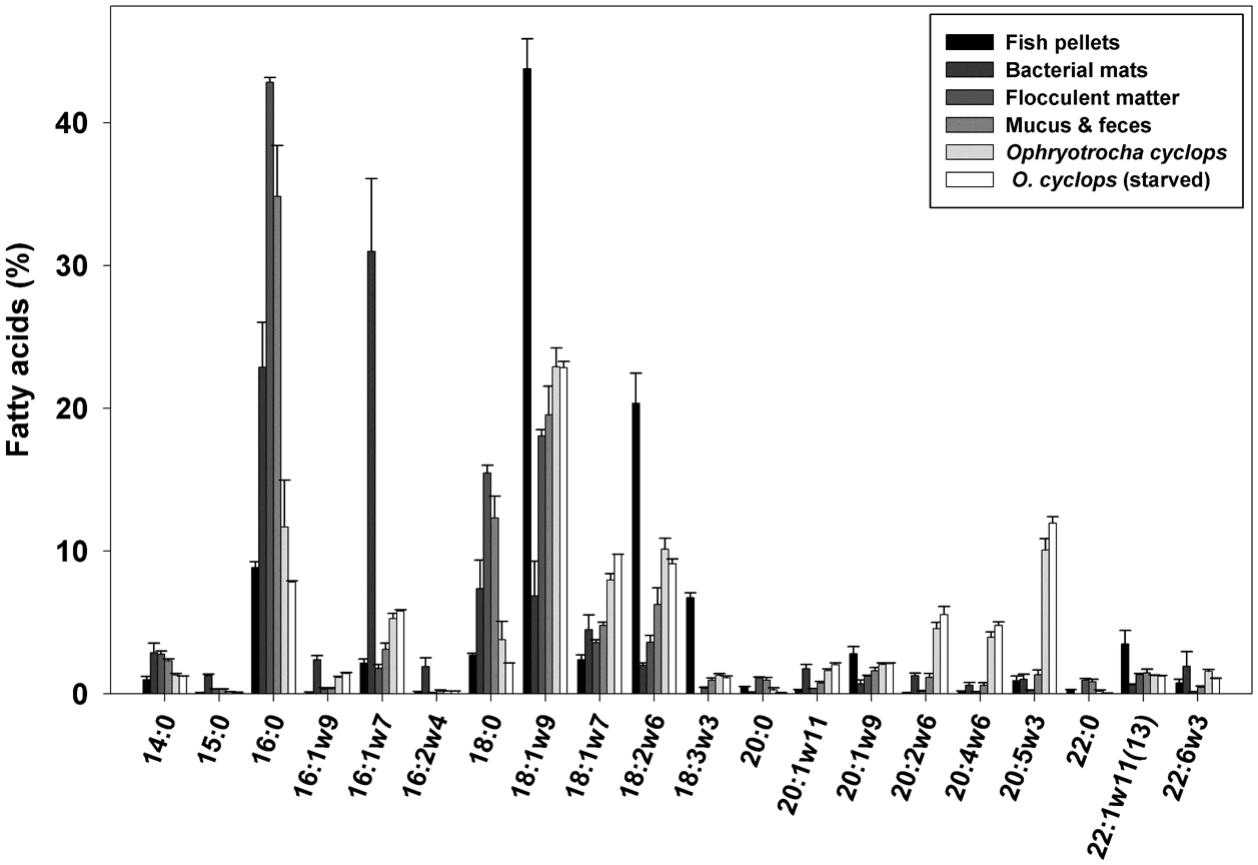
Fatty Acid Composition (% of total FA) of Samples from the Aquaculture Site. Only FA contributing to more than 1% of a sample’s composition are considered here. Error bars indicate SE.

**Fig 3:**
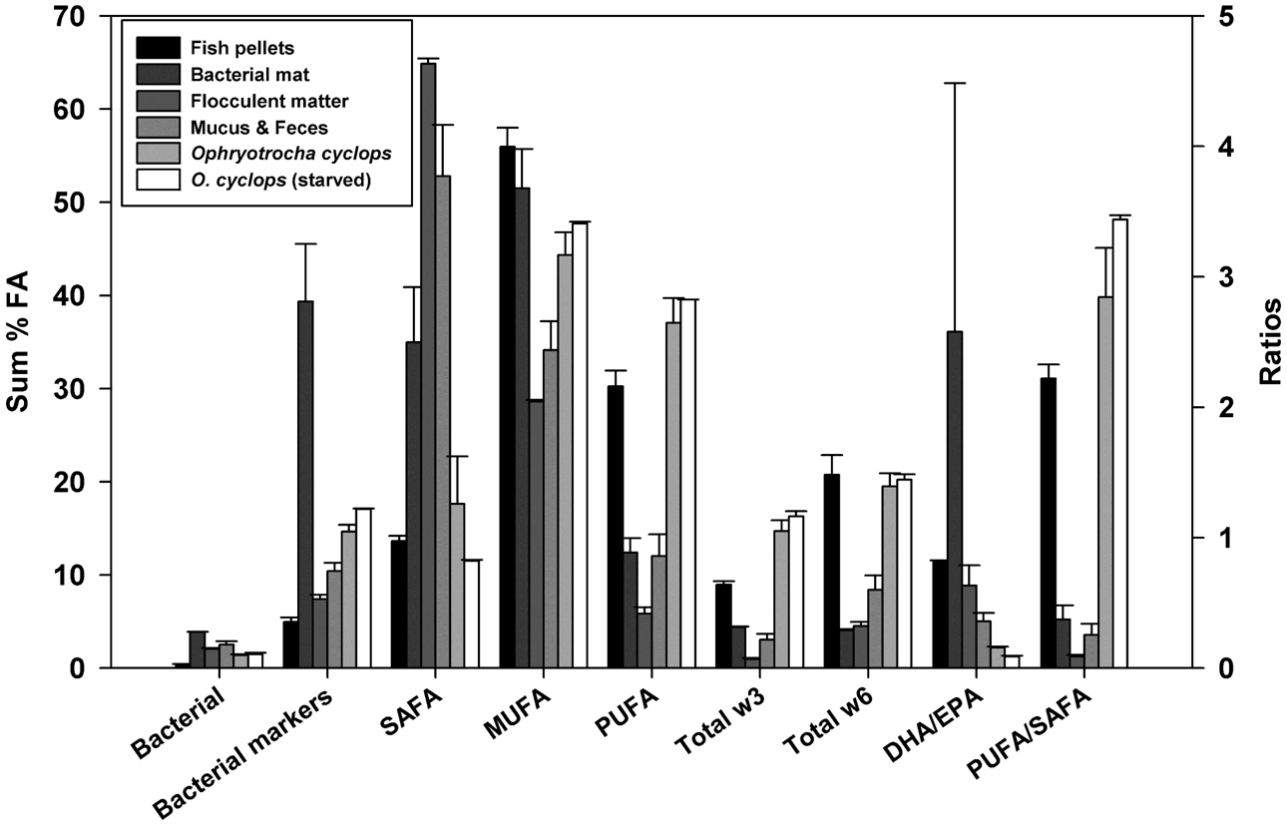
Fatty Acid Classes (% total FA) and ratios (DHA/EPA, PUFA/SAFA) of Samples Collected at the Aquaculture Site. Error bars indicate SE.

Fish pellets (n = 3) contained 157.6 ± 6.5 mg.g^-1^ (16% of the total dry mass) of lipids, consisting of 85.1 ± 0.8% TAG and between 2 to 4% each of FFA, ST, AMPL and PL (Fig 1). Fish pellet FA composition was dominated by MUFA (56.0 ± 2.0%, mainly 18:1ω9), then PUFA (30.2 ± 1.7%) and SAFA (13.6 ± 0.6%) (Figs 2 and 3). Other FA of note were 18:2ω6 (linoleic acid, 20%), 16:0 (palmitic acid, 9%), 18:3ω3 (8%) and 22:1ω11 (4%). Interestingly, EPA, AA, DHA and 20:2ω6 represent on average less than 1% of the total FA composition of fish pellets. The PUFA/SAFA ratio is also the greatest among the sources analyzed.

Bacterial samples (n = 2) had a lipid content of 43.7 ± 24.3 mg.g^-1^ (4% of the total dry mass). Predominant lipid classes consisted of PL (43.5 ± 43.5%, but present in one of the samples), TAG (16.2 ± 16.2%), FFA (26.9 ± 13.9%) and AMPL (13.4 ± 13.4%) (Fig 1). The main FA classes within bacterial samples were MUFA (51.5 ± 4.2%), SAFA (35.0 ± 5.9%) and PUFA (12.4 ± 1.5%); the category of bacterial markers including ω7 FA was well represented (39.3 ± 6.2%) (Figs 2 and 3). The main MUFA were 16:1ω7 (31.0 ± 5.1%), 18:1ω9 (6.9 ± 2.4%) and 18:1ω7 (4.5 ± 1.0%) and the main saturated FA were 16:0 (22.9 ± 3.1%) and 18:0 (7.4 ± 2.0%). Variation between the two bacterial replicates was considerable, particularly for lipids. Both ω3 and ω6 FA represent less than 4.5% of the total FA composition in the bacterial mat samples. On average, EPA, AA, 20:2ω6 and DHA each formed less than 1.5% of the total FA pool. The bacterial sample had the highest EPA/DHA ratio among the sources and organisms analyzed.

Flocculent matter (n = 3) contained 57 ± 24.8 mg.g^-1^ (6% of the total dry mass) of lipids, mainly composed of PL (28.1 ± 1.0%), ST (20.5 ± 0.6%), TAG (18.0 ± 3.3%), FFA (18.0 ± 1.7%) and AMPL (13.3 ± 4.4%) (Fig 1). Flocculent matter was rich in SAFA (64.9 ± 0.6%; mostly composed of palmitic acid and 18:0). The MUFA fraction (predominantly 18:1ω9) represented 28.6 ± 0.2% of the total FA composition, and the PUFA fraction was relatively low, at 5.8 ± 0.7% (Figs 2 and 3). The bacterial marker including ω7 class formed a small percentage (about 7.4 ± 0.4%) of the total FA in flocculent matter. A relatively high proportion of ω6 FA (4.5 ± 0.4%) was found in the flocculent matter, with a lower proportion of ω3 FA (1.0 ± 0.1%). The EPA, AA, 20:2ω6 and DHA comprised less than 0.3% of the total FA composition of the flocculent matter.

### Lipid and fatty acid composition of *Ophryotrocha cyclops*

When considering together both freshly-collected and laboratory maintained individuals, *Ophryotrocha cyclops* (n = 10) contained 130 ± 20 mg.g^-1^ (13% of the total dry mass) lipids, consisting of 47.2 ± 2.9% TAG, 31.3 ± 3.3% PL, 11.4 ± 0.9 ST, 5.1 ± 0.3% FFA and 3.1 ± 0.3% AMPL. Also present were HC, EE and GE, representing less than 1% of the total lipid composition (Fig 1). The FA content of worms was mostly composed of MUFA (45.0 ± 2.0%, mainly 18:1ω9 and 18:1ω7) and PUFA (37.5 ± 2.2%), followed by SAFA (16.4 ± 4.1%, dominated by 16:0). The FA classes of bacterial marker including ω7, and of ω3 FA (mostly EPA) represented 15.1 ± 0.7% and 15.0 ± 1.0% of the FA pool, respectively (Figs 2 and 3). The notable PUFA were 18:2ω6 (9.9 ± 0.6%), 20:2ω6 (4.8 ± 0.4%), and AA (4.1 ± 0.3%) (Figs 2 and 3). The PUFA/SAFA ratio is higher than in other analyzed samples (>3) whereas the DHA/EPA ratio is quite low (<0.2).

Maintaining *Ophryotrocha cyclops* in the laboratory for different periods (2 or 13 days) led to minor differences in their lipid and FA content and composition. Total lipid content over 2 weeks of laboratory maintenance decreased overall: 13.8 ± 2.3% in freshly collected worms *vs* 9.4 ± 0.7% after starvation. Little variation was observed when comparing each lipid class (proportion and quantity) for all classes except ethyl ketone which was present only in starved worms in highly variable concentrations (0.4 ± 0.4%). Generally, proportions of DHA, 20:1ω9, 18:3ω3, 16:2ω6 were higher, and proportions of EPA, AA, 20:2ω6, 20:1ω11, 18:1ω7, 16:1ω7 and 16:0 were lower in freshly collected worms than in those maintained for 13 days (Fig 2).

### Lipid and fatty acid composition of *Ophryotrocha cyclops* mucus and feces

Very small samples (n = 4) of *Ophryotrocha cyclops* mucus and feces were collected; the dry weight of these samples was below the detection limit of the balance we used. Mucus and feces are mostly composed of the following lipid classes: FFA (38.0 ± 10.3%), TAG (22.0 ± 3.2%), ST (17.1 ± 5.0%), PL 13.1 ± 13.1% (in one sample only) and AMPL (4.8 ± 1.9%) (Fig 1). The FA composition of the mucus and feces samples was dominated by SAFA (52.8 ± 5.5%; mainly 16:0 and 18:0) and MUFA (34.1 ± 3.1%, mainly 18:1ω9, with some 18:1ω7 and 16:1ω7), followed by PUFA (12.0 ± 2.3%, dominated by 18:2ω6). The category of bacterial marker including ω7 FA formed 10.4 ± 1.8% of the mucus and feces FA pool.

### Comparison of food source and consumer fatty acid composition

**Fig 4:**
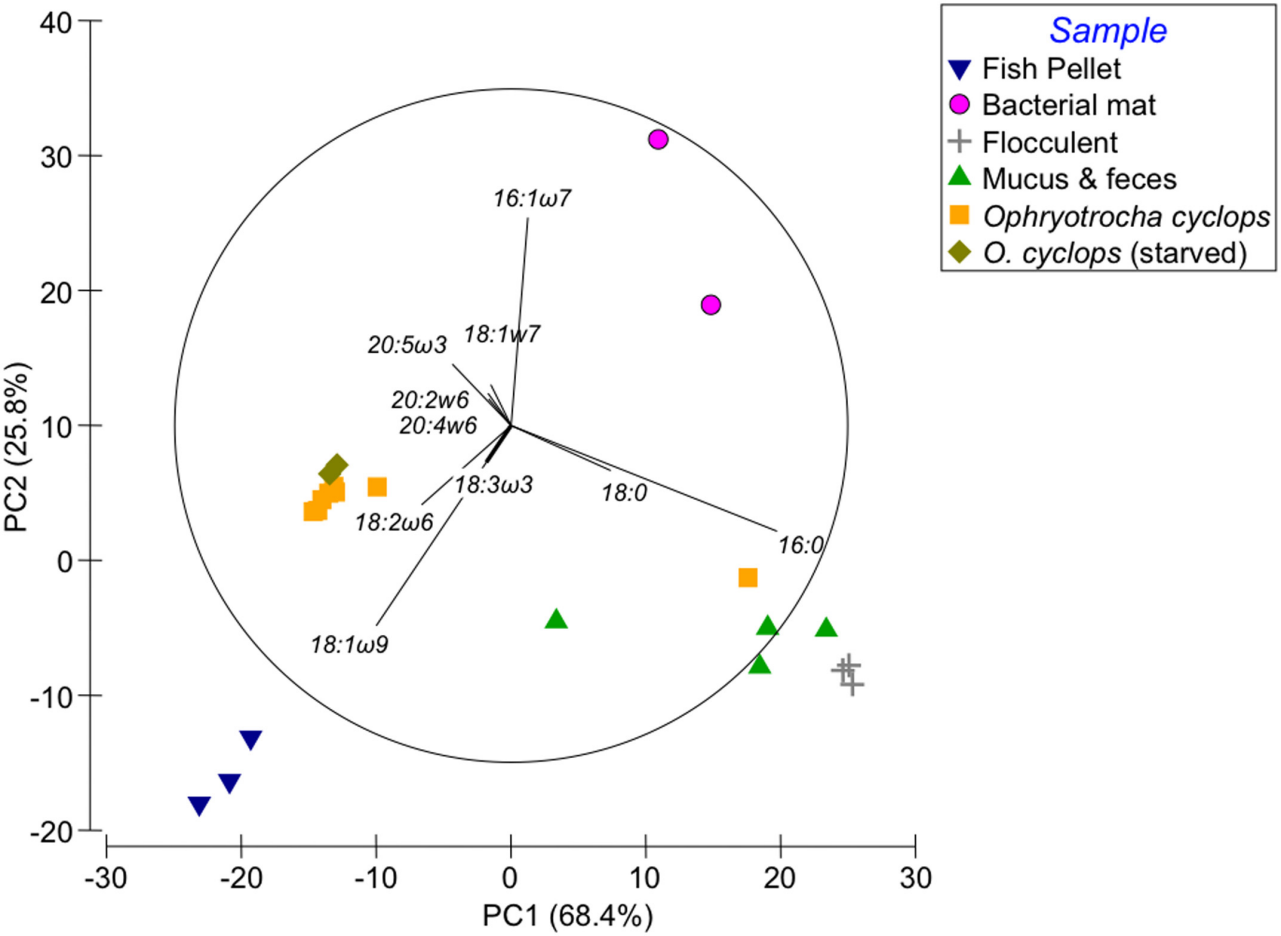
Principal Components Analysis. PCA was run on untransformed FA data (for correlations up to 0.1) and individuals have been superimposed on the figure.

The PCA separated all the sample types according to their FA composition, with replicates of each type grouped together (Fig 4). The first principal component explained 68.4% of the sample distribution and was mainly driven by the proportions of 16:0, 18:1ω9, 18:0, and 18:2ω6 (in relative importance). The second principal component explained 25.8% of the variability and was mainly driven by 16:1ω7 and 18:1ω9. Annelids, both those freshly collected and those experiencing starvation, were grouped together by their FA composition of 18:2ω6, 18:3ω3, EPA, 20:2ω6; 18:1ω7, AA and 18:1ω9, with the exception of a single sample that grouped with feces and mucus samples due to its high SAFA content (Fig 4). The fish pellet replicates were similar in composition, mainly due to their proportion of 18:1ω9. Bacterial mats, on the other hand, were more variable; they were aggregated mainly because of their 16:1ω7 content. Flocculent matter and mucus/feces samples were similar (with the exception of a single mucus and feces sample) due to their proportion of 16:0 and 18:0.

The PERMANOVA confirmed a significant difference between all samples (Pseudo F = 21.39, *p* = 0.001 with Monte-Carlo, 9999 permutations, Table 2), The PERMANOVA showed that samples within each group are similar, but that there are significant differences between groups, except for annelids before and after starvation. The SIMPER analysis (data not shown) revealed that fish pellets differed from bacterial mats: the former have a higher content in 18:1ω9 [27% dissimilarity (DS)] and 18:2ω6 (13% DS), a lower content in 16:1ω7 (21% DS) and 16:0 (10% DS), and contain 18:3ω3 (5% DS). Compared to flocculent matter, fish pellets have lower proportions of SAFA 16:0 (31% DS) and 18:0 (12% DS) and a higher content in 18:1ω9 (23% DS) and 18:2ω6 (by 15% DS). Bacterial mats mainly differ from flocculent matter by higher proportions of 16:1ω7 (31% DS) and lower proportions of palmitic acid (21% DS), 18:1ω9 (12% DS) and 18:0 (8% DS). *Ophyotrocha cyclops* differed from the other sources and from its own mucus/feces, but starvation did not affect their FA composition (the main, albeit weak, difference was a higher proportion of 16:0 in the freshly collected individuals). Overall, *O*. *cyclops* differs less from fish pellets than from other sources (Fig 4 and Table 2); *O*. *cyclops* and fish pellets differ by a higher content of 18:1ω9 and 18:2ω6 in the pellets and a prevalence of EPA and 18:1ω7 in the worms. Worms differ from flocculent matter by their lower SAFA content and greater proportion of EPA, and from bacterial mats by their lower 16:1ω7 and higher 18:1ω9, EPA and 18:2ω6 contents. Notably, 18:3ω3 is absent from the bacterial mat but present in worms, explaining its importance in the PCA. The mucus/feces samples were similar in composition to flocculent matter and the difference between the two is not driven by a particular FA composition but by their level of abundance. Mucus/feces differed from worms due to the higher level of SAFA (16:0 and 18:0, respectively 30 and 11% DS) and lower EPA content (11% DS) in mucus/feces, with EPA forming less than 1.5% of the total FA in mucus and 10% of total FA in worms.

**Table 2:** PERMANOVA Results. Pairwise average similarity within and between the samples based on the total matrix of FA using Monte Carlo *p* values with 9999 permutations (Pseudo F = 21.39, *p* = 0.001).

	Mucus	Fish pellet	*O*. *cyclops*	*O*. *cyclops* (starved)	Bacterial mat	Flocculent matter
**Mucus**	**84.5**					
**Fish pellet**	54.4***	**90.7**				
***O*. *cyclops***	60.9**	58.3***	**85.4**			
***O*. *cyclops* (starved)**	56.0**	56.1***	89.0	**96.5**		
**Bacterial mat**	57.0**	30.8**	45.2***	42.8*	**77.4**	
**Flocculent matter**	85.4	44.8***	50.6***	45.1***	55.3**	**96.0**

*: *p* < 0.05,

**: *p* < 0.01,

***: *p* < 0.001;

otherwise not significant.

### Observation of the gut epithelium

**Fig 5:**
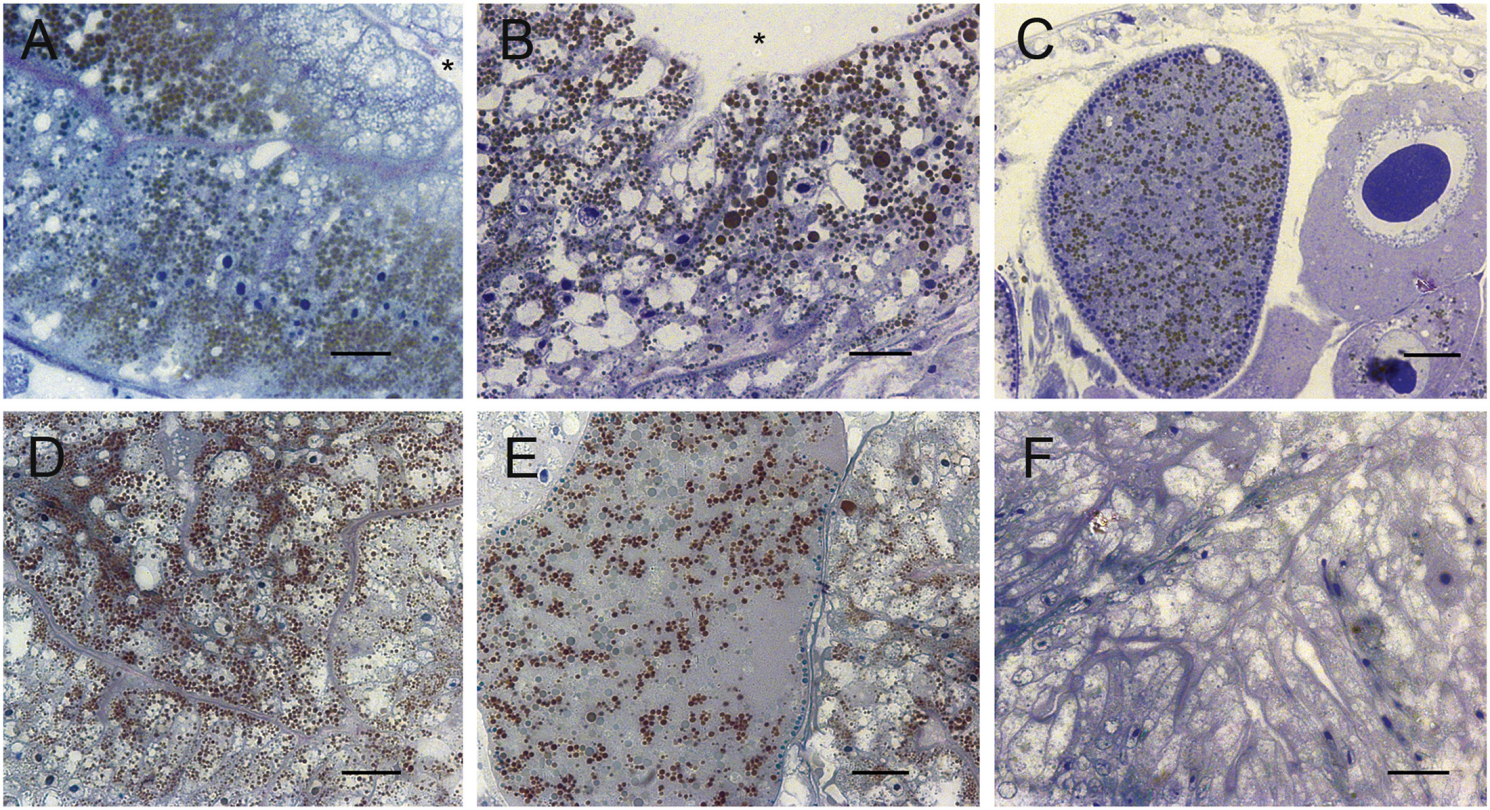
Transverse Sections of *Ophyotrocha cyclops*. Tissues were stained with osmium tetroxide and toluidine blue. The gut epithelium of specimens fixed upon collection (A), after 13 days (B), after one month (D) and after three months (F) are shown, as well as oocytes of the same specimens after 13 days (C) and one month (E). Lipid inclusions are stained in black with osmium tetroxide. The gut lumen is indicated by an asterisk. Scale bars: 40 μm.

A large quantity of lipid droplets (stained in black by osmium tetroxide) was observed in the medial part of the gut epithelium in freshly collected annelids as well as in those fixed after 13 days (Fig 5A and 5B). A slight reduction in the density and size of droplets took place over one month (Fig 5D). However after three months of laboratory maintenance without food, no visible droplets remained in the gut epithelium (Fig 5F). Lipid droplets were also evident in oocytes (when observed) of individuals fixed after 13 days and 1 month (Fig 5C and 5E).

## Discussion

In NL, *Beggiatoa*-like mats and *Ophryotrocha cyclops* are present at aquaculture sites, where they are usually associated with flocculent matter and surround the cage area with a high spatial and temporal variation according to site characteristics, distance from cage area, production cycle or season [49]. Within a single aquaculture site, we identified three main potential food sources available to *O*. *cyclops*, all of which harbored distinct lipid and FA signatures as revealed by the multivariate analysis.

### Composition of potential food sources

Fish pellets are designed to maximize the growth of fish in a short period of time, providing all the nutrients (e.g. proteins, essential FA, minerals and vitamins) that fish need [42]. Manufacturers combine ingredients of different origin: fishmeal, fish and plant oils along with poultry and crustacean derived products [42]. The proportion of ingredients varies according to the manufacturer, and is optimized for the species and size of fish cultured, the time of the year (summer/winter), period of production [42], and cost. The FA present in the fish pellets analyzed were rich in ω3 and ω6 and had the same FA composition as reported in other studies (16:0, 18:1ω9, 20:1ω9 and 22:1ω11), but in different proportions [36–38]. Here, fish pellet FA consisted mainly of oleic acid 18:1ω9 (43.8%, likely from plant oil; see Table 5 of [42]), followed by 18:2ω6 (20%), 16:0 (palmitic acid) and 18:3ω3 (7%), the latter two being important components of terrestrial plant oil and poultry fat [42,50]. Although fish pellets designed for salmonids in cold regions usually have a high lipid content (between 18 to 40% [42]), we noted a relatively low lipid content in our pellets (approximately 16%), perhaps related to the near-harvest stage of salmon at our study site. Fish pellets were characterized by a high TAG content (85% of lipids); TAG are readily absorbed and are essential for salmon growth, especially in sub-Arctic regions [51,52]. In this study, fish pellets can be discriminated from other potential sources by: 1) the presence of sterols (absent from bacteria); 2) the high level of TAG, composed of 18:1ω9, 18:2ω6 and 18:3ω3; and 3) other FA such as 20:1ω9 and 22:1ω11 that are in higher proportion in fish pellets than in the bacterial mat or flocculent matter. The latter two FA are considered to be zooplankton markers [34,53] and may indicate the presence of copepod-consuming fish [33] in the pellets [42].

The filamentous bacteria that we cultured in the laboratory are likely representative of mat-forming bacteria present at aquaculture sites (we have no molecular confirmation of the identity of either bacteria from the culture or from the study site). The main FA within these bacterial samples were 16:1ω7 (30%), 16:0 (23%), 18:0 (7%), 18:1ω9 (7%) and 18:1ω7 (5%). The main FA in *Beggiatoa* mats from gas hydrate sites are 16:1ω7 (67%); 18:1ω7 (17%) and 16:0 (8%) [54] which correspond to the same main FA in our bacterial culture but in different proportions. 16:1ω7 and 18:1ω7 were suggested biomarkers of sulfur-oxidising bacteria [54]; these two FA are often used as bacterial biomarkers in trophic relationship studies ([33,41,55,56], Table 1). The bacterial mats in our study were characterized by the absence of sterols, low levels of TAG, and high proportions of PL and bacterial marker including ω7 FA (predominantly 16:1ω7), confirming their bacterial signature [33].

The flocculent matter collected in the net was in accordance with previous descriptions of surficial sediments underneath fish cages: a dark, ultrathin texture with a strong sulfur odor [35,36,57], corresponding to what we observed using video imaging. Our lipid and FA profiles confirm that flocculent matter is a mixture of degrading feces, fish pellets and bacteria as was suggested in a previous study using stable isotope analysis [27]. Notably, flocculent matter in our study was composed of approximately equal parts of TAG, ST, PL, AMPL and FFA: TAG is a storage product highly abundant in pellets; ST and PL are natural constituents of cells, whereas AMPL and FFA are the breakdown products of the PL and TAG, indicating the degradation of fish pellets. Moreover, a single flocculent matter replicate was the only sample in this study that contained alcohol (also recorded in sediments from finfish aquaculture sites [38]); along with SE/WE and EE, these compounds are associated with the degradation of zooplankton [53]. As in our flocculent matter, others have reported high concentrations of TAG, SAFA and FFA in surface sediments at aquaculture sites [34–36,38,58], and interpreted them as being the result of two processes: 1) the natural sedimentation of uneaten pellets and 2) the non-total assimilation of these pellet compounds by fish. Additionally, flocculent matter is dominated by SAFA (65%, marked by a high abundance of 16:0 and 18:0, generally indicative of degradation), and MUFA (30%, dominated by 18:1ω9, also prevailing in our fish pellets), which is characteristic of material in degradation as typical planktonic markers (DHA and EPA) are low in flocculent matter. In addition, flocculent matter likely sustains bacterial populations, as supported by the presence (9%) of bacterial marker including ω7 FA.

### Integration and transformation of food sources by annelids

Interestingly, and contrary to our expectations given the abundant oil droplets in their gut epithelial cells, *Ophryotrocha cyclops* are composed of only 13% lipids. On average, polychaetes have a 1–60% lipid composition [59–62], with concentrations varying according to species, food availability, seasonal and reproductive patterns [33,59,60]. The pattern of dominance of TAG, followed by PL, ST then FFA recorded herein is common in marine polychaetes [60] but differs in extreme or subarctic habitats such as hydrothermal vents or abyssal environments where PL are dominant and TAG are in low proportion [61,63,64]. The dominance of TAG might indicate that the worms are able to form high energy stores in this environment [33,65]. The presence of up to 10% sterols, associated with a high level of PUFA as reported here, are common in the architectural composition of cold-adapted species [61,63]. However, while the most common FA are in accordance with other polychaetes (16:0, 18:1ω9, 18:1ω7, 18:2ω6, EPA) [60–62], FA dominance patterns of *O*. *cyclops* diverge from those polychaetes (as well as from congeners from methane seeps [66]) given the dominance of 18:1ω9, followed by EPA, 18:2ω6 and 18:1ω7.

Like PUFA/SAFA [67], the 18:1ω9/18:1ω7 ratio is often used to detect carnivory [41]. The 18:1ω9/18:1ω7 ratio ranges between 2–3 for *Ophryotrocha cyclops*, suggesting a high influence of 18:1ω9 (which can be synthesized using 18:0 as a precursor, unlike 18:1ω7 which has a bacterial origin or results from the elongation of 16:1ω7) and a carnivorous behaviour; however, the prevalence of 18:1ω9 in our fish pellets precludes us from using this index. The presence of jaws in *O*. *cyclops* [18] suggests either carnivory or grazing on bacterial mats, rather than suspension-feeding.

As previously discussed, 18:1ω9 and 18:2ω6 are abundant FA in worms (combined FA content of ≈33%) and are considered to have originated from the fish pellets in this study (Fig 4); although all organisms can synthesize 18:1ω9, we consider that this abundant FA results from a direct transfer from fish pellets. Alternatively, worms would have had to ingest a very high quantity of plant-derived matter which is improbable based on their small size and the paucity of algae/seaweed available at the collection site. In similar NL ecosystems, the importance of 18:2ω6 and 18:3ω3 in deposited organic matter is considered to be linked with terrestrial and litter forest inputs [68]. At our study site (a hard-bottom fjord), terrestrial input is unlikely to be important based on distance to land, explaining the low abundance of these two FA in the flocculent matter.

Bacterial markers, notably ω7 FA (mostly 16:1ω7 and 18:1ω7) are relatively abundant in worms in this study. These results suggest that *Ophryotrocha cyclops* consumes bacteria [56] as previously highlighted [27]. Other dorvilleids were also reported to consume bacteria and archaea [21,66,69]. Bacterial biomarkers could also result from a symbiotic association with bacteria [70] but we observed no bacterial symbionts during our previous transmission electron microscope observations [27].

The similarity among replicates of *Ophryotrocha cyclops* was due to the fish pellet-derived FA content (18:1ω9 and 18:2ω6) and high PUFA content, consisting of C_20_ FA, particularly EPA (Fig 4). Interestingly, 20:2ω6, AA and EPA were abundant in *O*. *cyclops*, but not in the food sources examined. Primary producers usually metabolize 18:1ω9 and 18:2ω6, resulting in elongated ω3 and ω6 carbon chains (>C_20_, i.e. EPA, AA and 20:2ω6), while heterotrophic organisms obtain those products via feeding. Here, those elongated FA represent a low proportion of all food sources analyzed and do not explain the high proportion of AA, EPA and 20:2ω6 FA in *O*. *cyclops* [33]. The ω6 FA are abundant in the fish pellets and found in a similar proportion in the annelids, however ω3 are proportionally more important in the annelids than in the pellets. Dominant FA in the food sources are 18:1ω9, 18:2ω6, 18:3ω3, 16:0 and 18:1ω7 and lead us to explore 3 hypotheses to explain the high levels of AA, EPA and 20:2ω6 in *O*. *cyclops*. The first hypothesis is that EPA, 20:2ω6 and AA are obtained from the food and accumulated/sequestrated in the tissues with very little excreted in mucus and feces. The FA 20:2ω6 plays an important role in the physiology of polychaetes, ranging in concentration between 2–50% [41,61,62]: this FA was shown to be important in the growth of juveniles of opportunistic polychaetes such as *Capitella capitata* [71] and measured in high abundance in other polychaetes or organisms from NL [61]. However, EPA and AA were present in the body of another *Ophryotrocha* species although they were raised on food lacking in those FA [69]. The second hypothesis is that *O*. *cyclops* possesses enzymes allowing it to transform FA present in the food by desaturation/elongation, resulting in EPA, AA or 20:2ω6. For example, the round worm *Caenorhabditis elegans* has the *Fat-1* gene which allows it to produce an ω3 desaturase which converts ω6 into ω3 [72]. *Ophryotrocha cyclops* could transform excess 18:2ω6 into EPA or 20:2ω6, explaining their intermediate content in 18:2ω6 and its decrease during starvation. EPA may also have been elongated from 18:3ω3. Other studies have shown that various consumers may have the enzymatic capability to synthesize EPA, 20:2ω6 and AA directly and use 18:2ω6 and 18:3ω3 as intermediates [73]. At hydrothermal vents, polychaetes may elongate and desaturate C_18_ FA, specifically the bacteria-derived 18:1ω7, to produce the essential FAs EPA and AA [63]. Likewise, the AA, 20:2ω6 and EPA reported in our study may have been synthesized from bacteria-derived FA and fish pellet derived products. The third hypothesis involves a transfer of FA or intermediates from bacteria (intestinal microflora or other symbionts) as suggested in other worms [56,74].

In another *Ophryotrocha* species, FA profiles were independent from those of potential dietary sources, suggesting *de novo* synthesis, with precursors difficult to identify [69]. Another study coupling FA and isotopic analyses showed that some dorvilleids consume archaea, but that no archaean biomarkers are apparent in worm isotopic or FA profiles [63,66,69]. However in our study, the importance of 18:1ω9 and bacterial marker including ω7 fatty acids confirm that *O*. *cyclops* consume bulk organic matter derived from the farm and might indirectly or directly select bacterial mats or ingest microbes; *O*. *cyclops* were observed to consume bacterial mats [18].

Mucus and feces had a low lipid content, as in other polychaetes [75]. These samples represent excreted lipids and consist of an important fraction of FFA and, interestingly, a high fraction of TAG and PL. The main FA in mucus and feces are SAFA (16:0, 18:0) but MUFA (18:1ω9, 18:1ω7 and 18:1ω6) are also important. If the abundant 16 and 18-chain FA in feces and mucus samples are mainly feces-associated, it may be that these FA are so abundant in the worms’ diet that only a small fraction gets assimilated, with the balance being excreted. Interestingly, 18:1ω9 is abundant in flocculent matter, worms and mucus, suggesting that the worm assimilates this FA but does not elongate/transform it, and excretes excess amounts. The high abundance of 18:1ω9 remaining in the flocculent matter over one month after removal of fish from the site suggests a low biodegradability of this FA; this may be a concern for site recovery after a production cycle [37].

Freshly sampled worms had higher proportions of 22:6ω3, 20:1ω9, 18:3ω3, 16:2ω6, and lower EPA, AA, 20:2ω6, 20:1ω11, 18:1ω7, 16:1ω7 and 16:0 than starved worms (Fig 2). Given the low sample size in this study and the fact that worms were pooled, statistical comparisons of FA content between freshly collected and starved worms were not possible, but we nonetheless observed a high similarity between the two groups (Table 2 and Figs 1–3). Microscopic observations suggest that a 13-day starvation period (Fig 5) may not be sufficiently long to provoke a notable decrease in FA and lipid stores, explaining this similarity. It might be interesting to look into which FA or lipid classes are preferentially used for energy and their potential longevity given their large stores of TAG and essential C_20_ FA. After three months of starvation the epithelial gut showed no visible lipid inclusions but the dorvilleids were still alive; this species may have a great capacity of tolerating periods of starvation, possibly enhancing dispersal abilities in cold waters.

In summary, and as suggested previously [27], *Ophryotrocha cyclops* from this study had mainly consumed fish farm derived organic matter (degrading fish pellets and feces), with a dietary contribution from bacterial mats as well. Where OPC are abundant, they may play important roles in converting excess fish pellets into living biomass. Overall, *O*. *cyclops* lipid and FA profiles reveal their high quality as food for organisms at higher trophic levels (i.e. rich in ω3 and ω6 FA [33]). Investigating any potential consumers of *O*. *cyclops* at aquaculture sites would be useful in understanding the transformation of organic matter in these environments.
